# Effects of ambient particulate exposure on blood lipid levels in hypertension inpatients

**DOI:** 10.3389/fpubh.2023.1106852

**Published:** 2023-02-21

**Authors:** Yanfang Gao, Chenwei Li, Lei Huang, Kun Huang, Miao Guo, Xingye Zhou, Xiaokang Zhang

**Affiliations:** ^1^School of Public Health and Health Management, Gannan Medical University, Ganzhou, China; ^2^Key Laboratory of Prevention and Treatment of Cardiovascular and Cerebrovascular Diseases of Ministry of Education, Gannan Medical University, Ganzhou, China; ^3^Gannan Medical University First Affiliated Hospital, Ganzhou, China

**Keywords:** ambient particulate matter, blood lipid markers, hypertension, arteriosclerosis, generalized additive model (GAM)

## Abstract

**Background:**

With modernization development, multiple studies of atmospheric particulate matter exposure conducted in China have confirmed adverse cardiovascular health effects. However, there are few studies on the effect of particulate matter on blood lipid levels in patients with cardiovascular disease, especially in southern China. The purpose of this study was to investigate the association between short- and long-term exposure to ambient particulate matter and the levels of blood lipid markers in hypertension inpatients in Ganzhou, China.

**Methods:**

Data on admission lipid index testing for hypertension inpatients which were divided into those with and without arteriosclerosis disease were extracted from the hospital's big data center from January 1, 2016 to December 31, 2020, and air pollution and meteorology data were acquired from the China urban air quality real time release platform from January 1, 2015 to December 31, 2020 and climatic data center from January 1, 2016 to December 31, 2020, with data integrated according to patient admission dates. A semi-parametric generalized additive model (GAM) was established to calculate the association between ambient particulate matter and blood lipid markers in hypertension inpatients with different exposure time in 1 year.

**Results:**

Long-term exposure to particulate matter was associated with increased Lp(a) in three kinds of people, and with increased TC and decreased HDL-C in total hypertension and hypertension with arteriosclerosis. But particulate matter was associated with increased HDL-C for hypertension inpatients without arteriosclerosis, at the time of exposure in the present study. It is speculated that hypertension inpatients without arteriosclerosis has better statement than hypertension inpatients with arteriosclerosis on human lipid metabolism.

**Conclusion:**

Long-term exposure to ambient particulate matter is associated with adverse lipid profile changes in hypertension inpatients, especially those with arteriosclerosis. Ambient particulate matter may increase the risk of arteriosclerotic events in hypertensive patients.

## 1. Introduction

Air pollution has become an environmental risk factor that seriously affects health, bringing a huge economic burden of disease and health loss to society (1, 2). According to a Comment by the World Health Organization (WHO), the number of premature deaths caused by air pollution has exceeded 7 million each year (3). Among air pollutants, ambient particulate matter (fine particulate matter with aerodynamic diameter <2.5 μm [PM_2.5_] and respirable particulate matter with aerodynamic diameter <10 μm [PM_10_]) is widely recognized as an important toxic component of air pollution mixtures (4). Most studies have found that ambient particulate matter exposure can increase the incidence of cardiovascular disease events (5, 6), among which hypertension as a risk factor for most cardiovascular diseases has been confirmed by most studies to be related to the health hazard effects of ambient particulate matter exposure (7, 8).

Blood lipids are the general term for neutral fats (triglycerides) and lipids (phospholipids, glycolipids, sterols, steroids) in plasma (9). Adverse alterations in lipid levels in humans can cause deposition of lipids in the vessel wall, which in turn triggers vascular stiffening they are also recognized as a risk factor for cardio - and cerebrovascular disease (10–12), and play an important role in chronic diseases such as hypertension, myocardial infarction, and ischemic stroke (13–16). It has been found that long-term exposure to ambient particulate matter can change the lipid level in the population (17, 18), but there is no consistency about the lipid effects of air pollution described by different studies (19, 20).

Ganzhou City is located in the southern of China. It has a typical humid subtropical monsoon climate. The precipitation is concentrated in spring and summer, the climate is mild, the heat and rainfall are abundant, the duration of cold and hot air flow is short, and the frost-free period is long. In recent years, Ganzhou's rare earth mining industry, tourism, furniture construction and other industries have developed rapidly, driving the steady growth of the city's economy. With the influx of population, the continuous increase in traffic flow, and the urbanization, the urban vitality of Ganzhou has been greatly improved, but it has also increased pollution emissions, posing challenges to air governance. As the economy develops, there are no studies on associations between air pollution exposure and blood lipids in Ganzhou. Therefore, this study collected ambient particulate matter exposure concentrations in Ganzhou City and blood lipid detection data of hypertensive inpatients in a tertiary hospital, and explored the impacts of ambient particulate matter pollution on blood lipid levels in hypertensive populations.

## 2. Methods

### 2.1. Blood lipid markers in hypertensive hospitalized patients

The hospitalization records of cardiovascular medicine from January 1, 2016 to December 31, 2020 were extracted from the big data center of a tertiary hospital (two campuses) in Ganzhou City, including the patient's admission date, main diagnosis, age, gender, and biochemical tests. According to the 10th revised International Classification of Diseases (ICD-10), whose code is Hypertension (ICD-10: I10-I13), we screened the information of patients with “hypertension” as their discharge diagnosis and matched them with data of blood lipid markers collected during hospitalization. Included lipid markers included triglycerides (TG), total cholesterol (TC), high-density lipoprotein cholesterol (HDL-C), low-density lipoprotein cholesterol (LDL-C), and lipoprotein [Lp(a)]. Since LP(a) is skewed distribution data, we logarithmically transform LP(a). The hypertensive inpatients were divided into “hypertension with arteriosclerosis population” and “hypertension without arteriosclerosis population” according to whether other discharge diagnoses included arteriosclerosis. The sum of the two populations was called “total hypertension population.”

### 2.2. Air pollution and meteorology data of Ganzhou City

**Figure 1:**
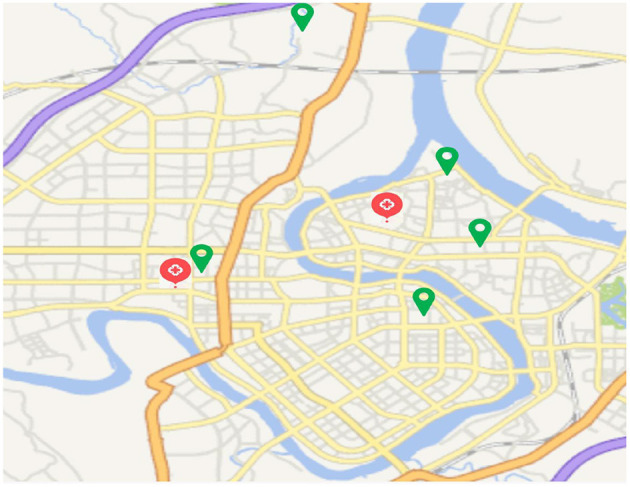
The locations of air pollutant data monitoring sites (green symbol) and hospital admissions (two campuses, red symbol).

The air pollutant concentration data of Ganzhou City from January 1, 2015 to December 31, 2020 were obtained on the China Urban Air Quality Real-time Release Platform. Calculate and sort out the 24-h average concentrations of five air pollutants such as PM_2.5_, PM_10_, carbon monoxide (CO), Nitrogen dioxide (NO_2_), and sulfur dioxide (SO_2_) and the daily maximum 8-h average ozone (O_3_) concentration. Air pollution exposure concentrations were matched according to the patients' hospitalization dates to generate a total of 15 air pollution concentration data series including L0, L0-6, L0-13, L0-29, L0-59, L0-89, L0-119, L0-149, L0-179, L0-209, L0-239, L0-269, L0-299, L0-329, and L0-359. Where L0 is equivalent to the same day exposure concentration of the respective air pollutant and L0-6 are equivalent to the moving average exposure concentrations at a lag of 6 days between hospital admissions for patients with hypertension. Meteorological data comes from the National Climate Data Center, which collects indicators such as the daily average temperature (°C) and relative humidity (%) in Ganzhou from January 1, 2016 to December 31, 2020. The locations of air pollutant data monitoring sites and hospital admissions (two campuses) are shown in Figure 1.

### 2.3. Statistical analysis

SPSS was used to describe the mean ± standard deviation (Mean ± SD) of blood lipid indexes in three groups of “hypertension with arteriosclerosis,” “hypertension without arteriosclerosis” and “total hypertension.” Age, gender and hospitalization date were used as covariates for covariance analysis to compare the differences in the mean concentrations of blood lipid indexes among the three groups. If differences were statistically significant, Bonferroni's method was used for multiple comparisons (21). The central and discrete trends of air pollution and meteorological data were described, and the differences in air pollution concentrations between adjacent years were compared using the Kruskal-Wallis test (22). Spearman correlation analysis was used to evaluate the associations between air pollutants and meteorological data (23).

R4.0.4 was used to establish a semi-parametric generalized additive model (GAM) (24, 25) to adjust the temperature, atmospheric relative humidity, age and gender of patients and balance the time trend to calculate the effect of changes in the unit concentration of air pollution on blood lipids in the study population. On this basis, choose the lag time period in which the ambient particulate matter has the strongest correlation with the blood lipid concentration of the population in the lag of 1 year to establish a dual-pollutant model, and observe the impacts of ambient particulate matter exposure on the blood lipid concentration of the three groups of people after adjusting for the concentration of gaseous pollutants. The partial regression coefficient and 95% confidence interval of the effect of ambient particulate matter on the concentration of blood lipid markers were obtained, and converted into the change value of blood lipid concentration by exp[ (β × 10)-1] × 100. That is, the corresponding change in blood lipid concentration for every 10 μg/m3 increase in PM_2.5_ or PM_10_ concentration, and its 95% confidence interval.

## 3. Results

### 3.1. Characteristics of lipid markers in study subjects

Among the hypertensive hospitalized patients, 2,269 cases of TC and TG were matched respectively, among which 685 cases had arteriosclerosis, 2,268 cases of HDL-C had 685 cases of arteriosclerosis, 2,266 cases of LDL-C had 685 cases of arteriosclerosis, 2,241 cases of Lp(a) of which 674 had arteriosclerosis. As shown in Table 1, the mean values of the five lipid markers in the total hypertensive population were: TG 1.74 mmol/l, TC 4.45 mmol/l, HDL-C 1.1 mmol/l, LDL-C 2.69 mmol/l, and LP (a) 1.81 μmol/L. There were no significant differences in lipid concentrations among the three groups by covariance analysis.

**Table 1:** General lipid profile of the study population.

	**Total hypertension**		**Hypertension with arteriosclerosis**		**Hypertension without arteriosclerosis**		***P***
	***N***	**Mean (SD)**	***N***	**Mean (SD)**	***N***	**Mean (SD)**	
TG (mmol/L)	2,269	1.74 (1.53)	685	1.66 (1.31)	1,584	1.78 (1.62)	1.000
TC (mmol/L)	2,269	4.45 (7.23)	685	4.33 (1.10)	1,584	4.51 (8.63)	0.846
HDL-C (mmol/L)	2,268	1.1 (0.29)	685	1.08 (0.29)	1,583	1.05 (0.29)	0.754
LDL-C (mmol/L)	2,266	2.69 (0.89)	685	2.71 (0.91)	1,581	2.68 (0.88)	0.147
Lp(a) (μmol/L)	2,241	1.81 (0.62)	674	1.83 (0.63)	1,567	1.81 (0.62)	0.772

### 3.2. Characteristics of air pollution and meteorological data

**Figure 2:**
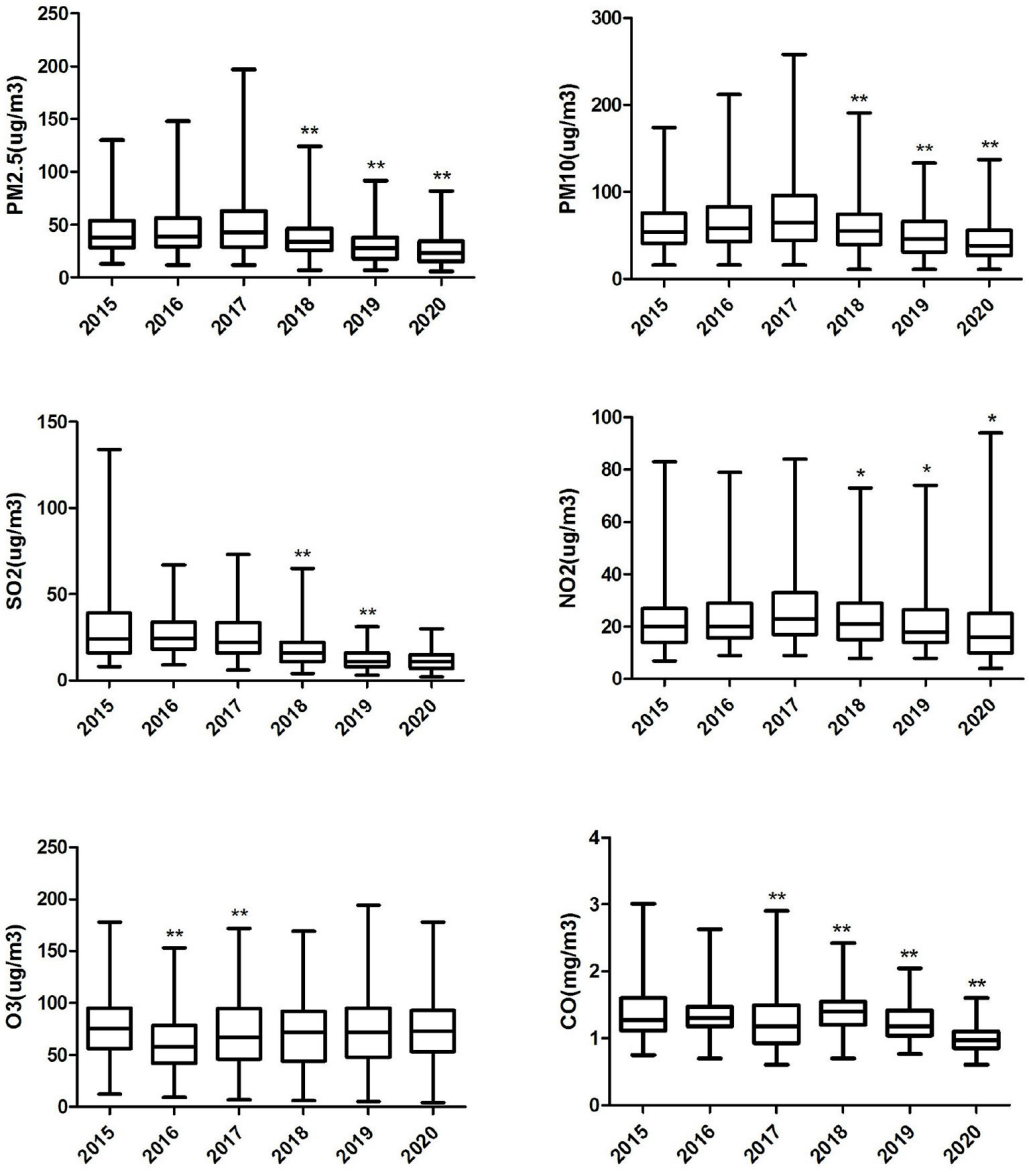
Box plot of annual air pollution concentration in Ganzhou City, 2015–2020. “*” Differences between pollutant concentrations in the marker year and the previous year 0.001≤*P*< 0.05. “**” Differences between pollutant concentrations in the marker year and the previous year *P* < 0.001.

Table 2 shows that the average concentrations of CO, NO_2_, O_3_, PM_10_, PM_2.5_, and SO_2_ are 1.27 mg/m^3^, 23.171 μg/m3, 70.80 μg/m3, 59.89 μg/m3, 38.17 μg/m3 and 20.94 μg/m3, respectively. The daily average temperature and relative humidity were 19.72°C and 74.37%, respectively. Figure 2 shows the change in the annual average concentration of air pollutants from 2015 to 2020 compared with the previous year. From 2018 to 2020, the annual average concentrations of PM_2.5_, PM_10_, NO_2_ and CO showed a clear downward trend year by year. The annual mean concentration of SO_2_ decreased significantly in 2018 and 2019. The annual average concentration of O_3_ dropped significantly in 2016 and again in 2017 was significantly higher than the previous year.

**Table 2:** General situation of air pollutant concentration in 2015–2020 and temperature and humidity in 2016–2020.

**Exposure**	***N***	**Mean (SD)**	**Median**	**Minimum**	**Maximum**	**IQR**
**Air pollution** ^a^						
CO (mg/m3)	2,192	1.27 (0.34)	1.23	0.6	3.01	0.45
NO_2_ (μg/m3)	2,192	23.17 (13.06)	20	4	94	14
O_3_ (μg/m3)	2192	70.80 (32.05)	68	5	194	43
PM_10_ (μg/m3)	2,192	59.89 (32.75)	52	11	258	39
PM_2.5_ (μg/m3)	2,192	38.17 (20.62)	34	6	197	24
SO_2_ (μg/m3)	2,192	20.94 (13.56)	17	2	134	15
**Atmosphere**						
Temperature (°C)	1,827	19.72 (8.07)	21	0	32	4
Humidity (%)	1,827	74.37 (12.19)	74	35	99	19

^a^Average air pollution concentration of five monitoring points in the central urban area of Ganzhou.

As shown in Table 3, positive correlations were observed among all air pollutants except O_3_, for which ambient particulate matter had stronger correlations with NO_2_ and SO_2_. There was a negative correlation between both air temperature and humidity and with ambient particulate matter, but positive correlations between air temperature and O_3_ or SO_2._as well as humidity and CO were observed.

**Table 3:** Spearman correlation analysis between air pollutants and meteorological factors in Ganzhou City.

	**CO**	**NO_2_**	**O_3_**	**PM_10_**	**PM_2.5_**	**SO_2_**	**Temperature**
NO_2_	0.401^**^						
O_3_	−0.182^**^	−0.095^**^					
PM_10_	0.386^**^	0.655^**^	0.327^**^				
PM_2.5_	0.445^**^	0.618^**^	0.257^**^	0.958^**^			
SO_2_	0.292^**^	0.510^**^	0.220^**^	0.705^**^	0.687^**^		
Temperature	−0.320^**^	−0.394^**^	0.370^**^	−0.126^**^	−0.211^**^	0.066^**^	
Humidity	0.210^*^	−0.073^**^	−0.642^**^	−0.359^**^	−0.228^**^	−0.293^**^	−0.316^**^

^**^p < 0.001. ^*^0.001 ≤ P < 0.05.

### 3.3. Blood lipids effects on hypertension inpatients with arteriosclerosis

**Figure 3:**
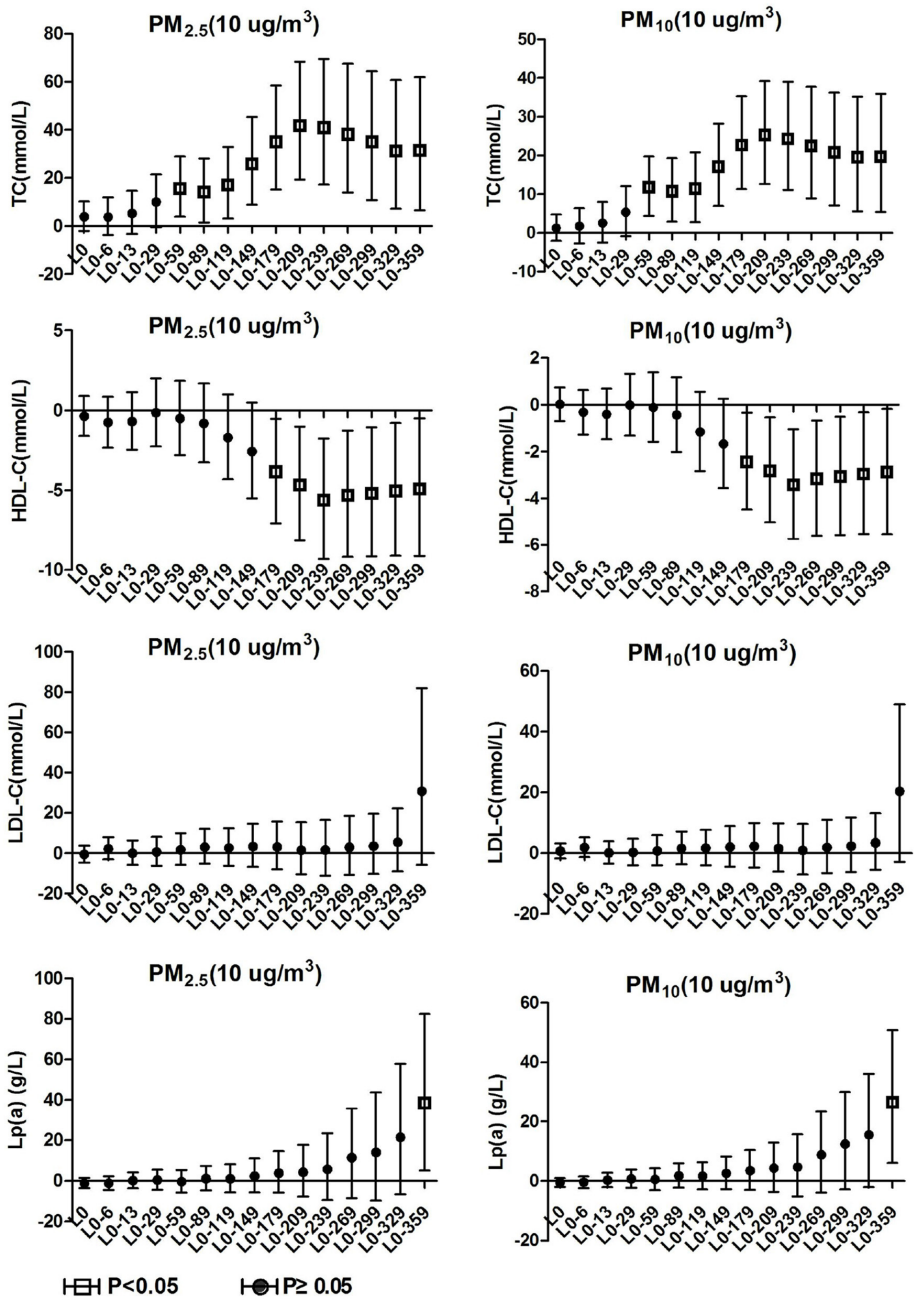
Effect of PM_2.5_ and PM_10_ on blood lipid in hypertension inpatients with arteriosclerosis.

As shown in Figure 3, it was observed in the hypertensive population with arteriosclerosis that PM_2.5_ and PM_10_ maintained a significant positive correlation with TC starting from lag0-59. At lag0-209, every 10 μg/m3 increase in PM_2.5_ and PM_10_ caused 41.8% (95%CI: 19.39, 68.42) and 25.25% (95%CI: 12.67, 39.23) increases in TC concentration, respectively. From lag 0-179, HDL-C maintained a long negative correlation with PM_2.5_ and PM_10_. At lag 0-239, ambient particulate matter had the most significant effect on HDL-C, and every 10μg/m3 increase in PM_2.5_ and PM_10_ caused 5.61% (95%CI: 1.76, 9.31) and 3.42 (95%CI: 1.04, 5.74) decrease in HDL-C, respectively. The effects of PM_2.5_ and PM_10_ on LDL-C and TG in hospitalized patients with hypertension and arteriosclerosis were not observed with a lag of 359 days in the moving average of ambient particulate matter exposure. Significant positive correlations between PM_2.5_ and PM_10_ and Lp(a) were observed only at lag0-359. Lp(a) increased by 38.52% (95%CI: 5.09, 82.59) and 26.47% (95%CI: 6.03, 50.84) for each 10 μg/m3 increase in PM_2.5_ and PM_10_, respectively.

### 3.4. Blood lipids effects on hypertension inpatients without arteriosclerosis

**Figure 4:**
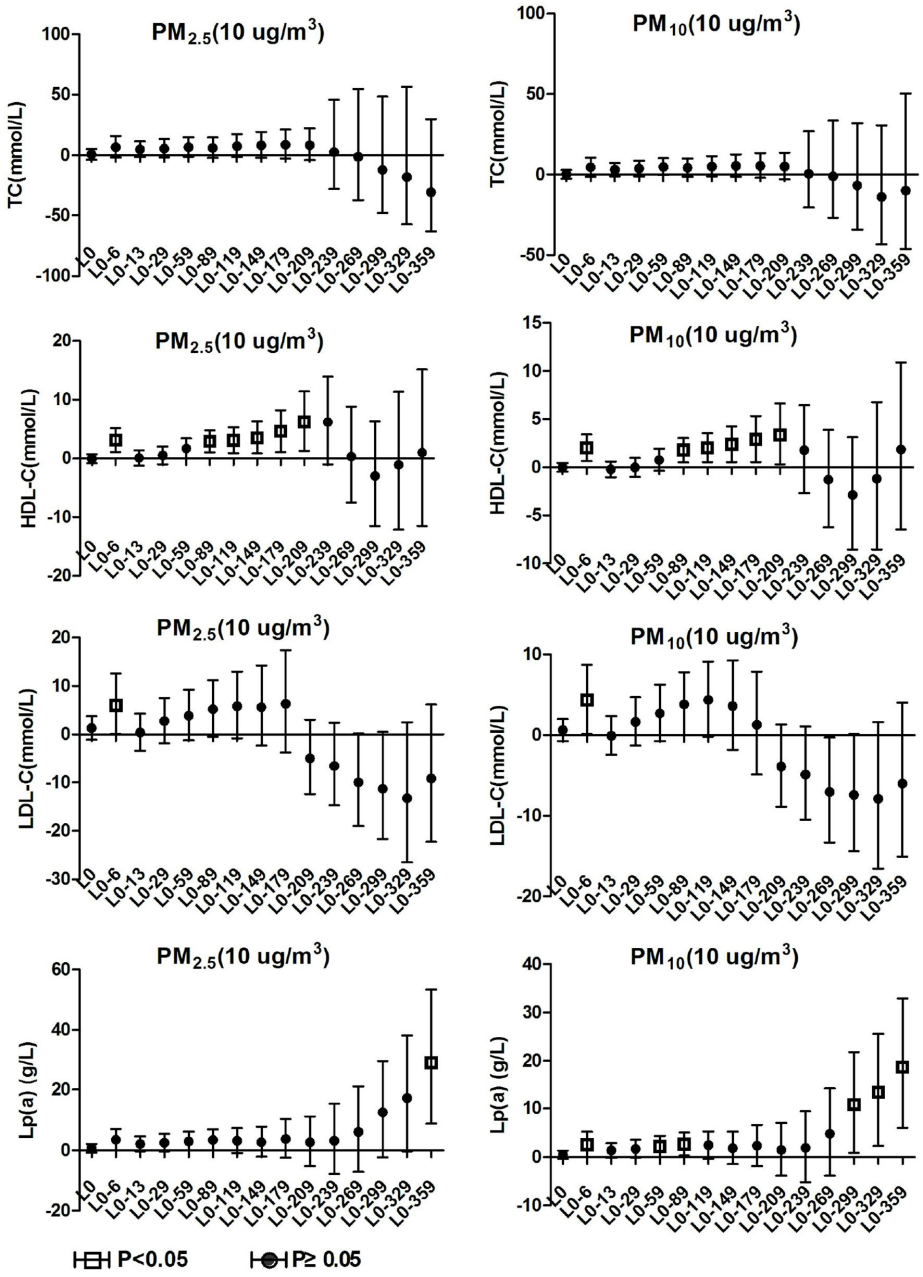
Effect of PM_2.5_ and PM_10_ on blood lipid in hypertension inpatients without arteriosclerosis.

TC and TG in hypertension inpatients without arteriosclerosis were not associated with ambient particulate matter exposure during the study period. As show in Figure 4, HDL-C and LDL-C were both significantly positively correlated with ambient particulate matter at lag0-6, and HDL-C showed a positive correlation with ambient particulate matter again at lag0-89 to lag0-209. During the study period, PM_2.5_ was positively correlated with Lp(a) concentration in hypertensive population without arteriosclerosis only at lag 0-359. Lp(a) increased by 29.19% (95%CI: 8.75, 53.46) for every 10 μg/m3 increase in PM_2.5_. PM_10_ showed a positive correlation with Lp(a) concentrations in this population at various time periods, including lag0-6, lag0-59 to lag0-89, and lag0-299 days later. These results revealed that hypertension inpatients without arteriosclerosis has better statement than hypertension inpatients with arteriosclerosis on human lipid metabolism.

### 3.5. Blood lipids effects on total hypertension inpatients

**Figure 5:**
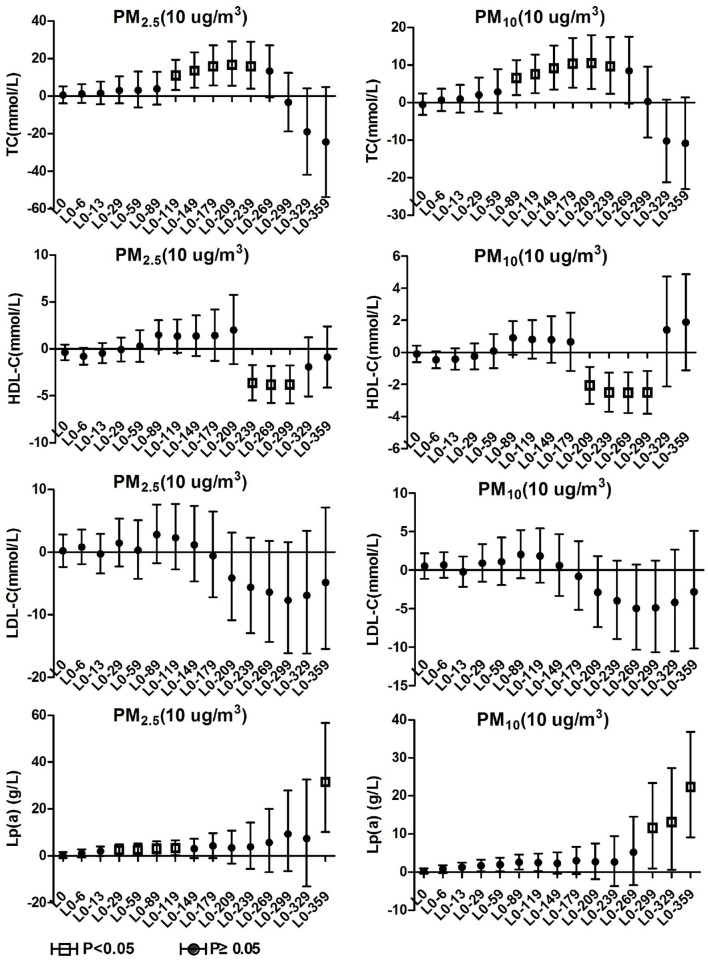
Effect of PM_2.5_ and PM_10_ on blood lipid in total hypertensive inpatients.

As show in Figure 5, PM_2.5_ and PM_10_ were significantly positively correlated with TC levels in total hypertensive patients at lag0-119 to lag0-239 and lag0-89 to lag0-239, respectively. For every 10 μg/m3 increase in PM_2.5_ and PM_10_ at lag 0-209, TC in total hypertensive patients increased by 16.77% (95%CI: 5.52, 9.22) and 10.54% (95%CI: 3.60, 17.94), respectively. PM_2.5_ and PM_10_ exposures were positively correlated with Lp(a) concentrations in hypertensive patients from lag0-29 to lag0-119, the correlation disappeared at lag0-149, and then reappeared from lag0-359 and lag0-229, respectively. Lp(a) concentrations in patients with a 10μg/m3 increase in PM_2.5_ and PM_10_ at lag0-359 increased by 31.41% (95%CI: 10.16, 56.76) and 22.25% (95%CI: 9.16, 36.91), respectively. PM_2.5_ and PM_10_ were negatively correlated with HDL-C levels in this population at lag0-239 to lag0-229 and lag0-209 to lag0-229, respectively. For each 10 μg/m3 increase in PM_2.5_ and PM_10_ at lag 0-239, HDL-C concentrations in patients decreased by 3.63% (95%CI: 1.71, 5.50) and 2.49% (95%CI: 1.26, 3.69), respectively. No correlation was observed between ambient particulate matter exposure and LDL-C and TG in the total hypertensive population.

### 3.6. Blood lipids effects after adjusting for gaseous pollutants

**Figure 6:**
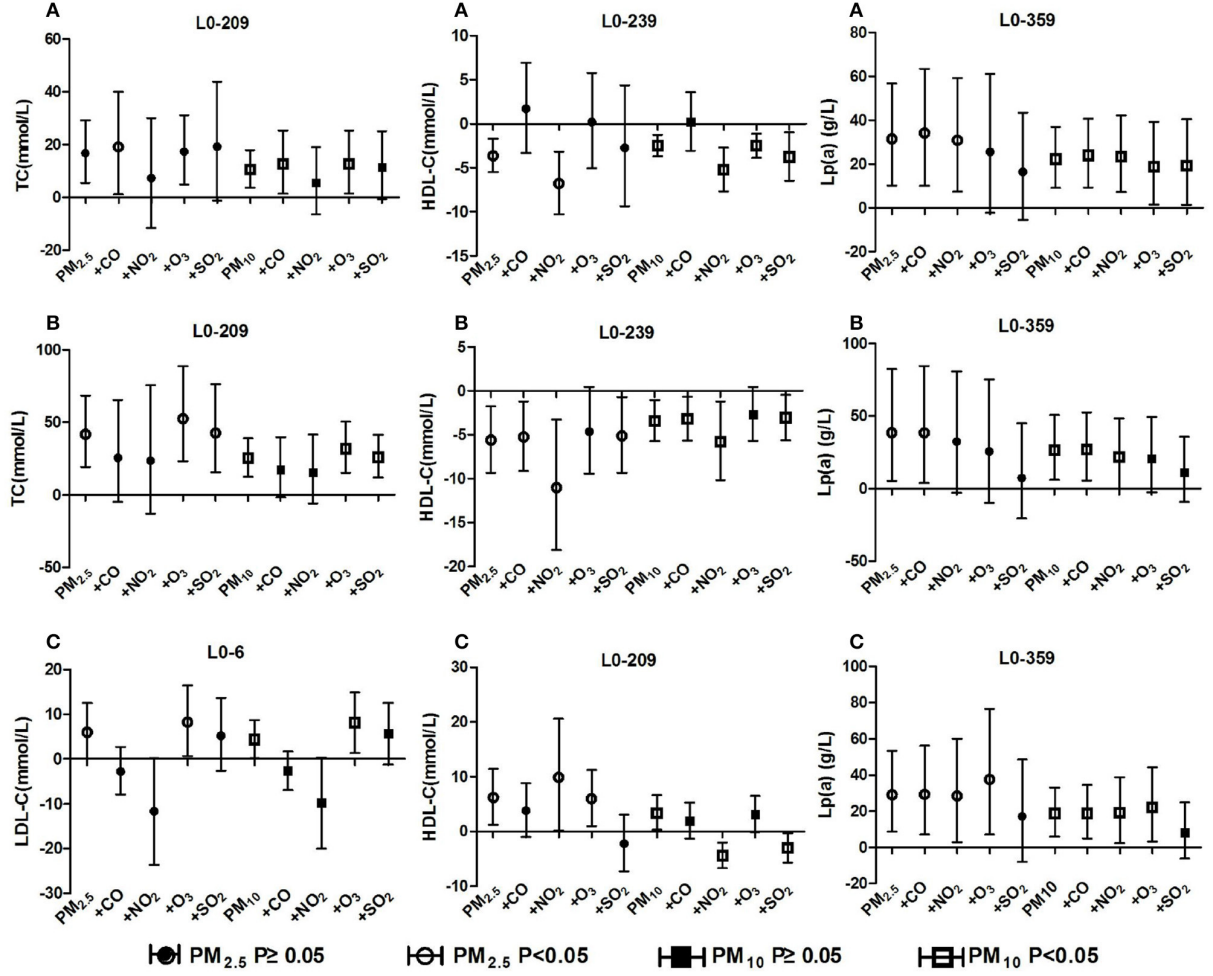
Effect of ambient particulate matter on blood lipid after adjusting for gaseous pollutants. **(A)** Effect of ambient particulate matter on blood lipid after adjusting for gaseous pollutants in total hypertensive inpatients, **(B)** Effect of ambient particulate matter on blood lipid after adjusting for gaseous pollutants in hypertension inpatients with arteriosclerosis, **(C)** Effect of ambient particulate matter on blood lipid after adjusting for gaseous pollutants in hypertension inpatients without arteriosclerosis. +CO, +NO_2_, +O_3_, +SO_2_ two-pollutant model after adjusting for the concentration of CO, NO_2_, O_3_, and SO_2_ respectively.

The positive correlation between ambient particulate matter and TC in the population with total hypertension and hypertension with arteriosclerosis remained unchanged after adjusting for gaseous pollutants, but it disappeared significantly after adjusting for NO_2_ concentration. The positive correlation between particulate matter and Lp(a) of the three groups of people remained unchanged after adjusting for the effects of gaseous pollutants, but the significant effects of particulate matter disappeared after adjusting for SO_2_, O_3_ and NO_2_. In the total hypertensive population, ambient particulate matter was negatively correlated with HDL-C, and the correlation changed positively after CO adjustment. The correlation between PM_10_ and HDL-C changed from positive to negative after adjusting for NO_2_ and SO_2_ in the hypertensive population without arteriosclerosis. The positive correlation between ambient particulate matter and LDL-C levels in hypertensive patients without arteriosclerosis was reversed after adjusting for CO and NO_2_, but there was not significant. The detailed results are shown in Figure 6. These results revealed that long-term exposure to ambient particulate matter is associated with adverse lipid profile changes in hypertension inpatients, especially those with arteriosclerosis

## 4. Discussion

In recent years, Ganzhou has stepped up its air pollution control actions. Since 2017, the concentration of ambient particulate matter has decreased significantly year by year. Ganzhou City belongs to southern China and has not established a unified heating system. However, in the cold winter, residents will generally have individual combustion or energy-consuming heating behaviors, resulting in an increase in the emission of ambient particulate matter and a negative correlation between the concentration of particulate matter and meteorological factors. In addition, the low temperatures in winter may cause poor diffusion of pollutants in the air, leading to widespread peaks of pollutants in the cold season (26). Similar to most southern cities, the main sources of ambient particulate matter emissions in Ganzhou are construction dust and vehicle exhaust from urbanization. From 2015 to 2020, the average daily concentration of PM_10_ in Ganzhou was 59.89 μg/m3, which was higher than the first-level standard of the national ambient air quality standard (GB3095-2012), but lower than the second-level standard (the first-level standard was 40 μg/m3, and the second-level standard was 70 μg/m3). The average daily concentration of PM_2.5_ was 38.17 μg/m3, which was higher than the NAQS secondary standard (15 μg/m3 for the primary standard and 35 μg/m3 for the secondary standard).

Total cholesterol (TC) refers to the sum of cholesterol contained by all lipoproteins in the blood, including free cholesterol and cholesterol esters. Most of the cholesterol is mainly synthesized by the body itself and from food sources as a minor supplement. There are these lipid transfer proteins which are HDL-C, LDL-C, and very low-density lipoprotein cholesterol. High density lipoprotein (HDL) can uptake low-density lipoprotein, cholesterol, triglycerides, sedimented from the intimal lining of the vessel wall for excretion to the liver, which helps to resist the stiffening of blood vessels caused by hyperlipidemia (27) and maintain cardiovascular health (28–30). Low density lipoprotein (LDL) is a lipoprotein particle that carries cholesterol into tissue cells and carries cholesterol accumulation across the arterial wall to cause arteriosclerosis (31). Lipoprotein [Lp (a)] is synthesized by the liver and is a specialized cholesterol rich macromolecular lipoprotein that promotes atherosclerosis (32).

This study found differences in the effects of ambient particulate matter on TC, HDL-C, LDL-C, and Lp (a) content among hypertension with or without arteriosclerosis. Combined with the analysis of biochemical associations among lipid markers to identify differences in the associations of each lipid marker with ambient particulate matter among different populations, we raise several conjectures.

Ambient particulate matter exposure exhibited health hazard effects of increasing TC levels and decreasing HDL-C levels for both the hypertensive with arteriosclerosis population and the total hypertensive population. However, in the hypertensive without arteriosclerosis population, there are no association with TC levels was present but increasing HDL-C levels emerged with ambient particulate matter exposure. There was a similar trend for Lp (a) levels to increase with higher ambient particulate matter concentrations in the three groups. A positive association with short-term particulate matter exposure was observed only in people with hypertension without arteriosclerosis. It is speculated that ambient particulate matter exposure mainly cause increased cholesterol and lipoprotein synthesis and decreased high-density lipoprotein synthesis in hypertensive patients. But in patients without arteriosclerosis, the regulatory mechanisms of lipid homeostasis are normal, so that lipid transfer proteins such as HDL-C and LDL-C were temporarily increases to maintain stable total cholesterol levels. When cholesterol synthesis continues increasing with long-term exposure, beneficial HDL-C is more producted to metabolize excess cholesterol. Meanwhile LDL-C is inhibited temporarily to protect blood vessels. The phenomenon of compensatory increases in HDL-C and LDL-C with ambient particulate matter exposure has thus emerged in hypertensive population without vascular stiffening.

Long-term exposure to air pollution was found to be associated with increased TC and LP (a) levels in the elderly, obese adolescents and cardiovascular disease (CVD) patients in other studies on associations between air pollution and lipid concentrations (33–35), and additional reports showed that lower HDL-C levels were associated with particulate matter exposure (19, 34, 36). Two studies of the association between air pollution and blood lipids in adults in China both showed that particulate matter had a deleterious effect on blood lipid markers, and this effect was more pronounced in people who were overweight or obese (37). These studies are similar to the results of ambient particulate matter and lipid-related changes that we observed in hypertensive patients with arteriosclerosis and total hypertension. Particulate matter exposure may have a stronger effect on raising TC and lowering HDL-C in individuals already at high risk, a study of older U.S. men suggests (38). This corresponds to our findings and conjectures.

The cardiovascular harm of ambient particulate matter exposure has been widely confirmed, and it has been proposed that oxidative stress (39, 40), inflammatory response (41, 42), and DNA methylation (43, 44) are the main damage mechanisms. Dyslipidemia is the most sensitive metabolic risk factor to air pollution exposure (45). Ambient particulate matter can cause systemic inflammation and oxidative stress, inducing adverse lipid metabolism and oxidation (46). The inflammatory response further mediates a lipid compensatory response against the invasion of hazardous substances from ambient particulate matter and aids tissue repair. When the repair of injury is saturated by lipid compensatory responses, cascading repetitive stimulation of the inflammatory response enhances atherosclerotic lesion formation (47). Additionally ambient particulate matter can also lead to specific gene methylation related to lipid metabolism by reducing the activity of DNA methyltransferases (48). The above studies exhibited different mechanistic pathways by which ambient particulate matter altered blood lipids, which could directly provoke adverse lipid metabolism and also provoke lipid compensatory responses in the pre hazard period, corresponding to the differences in relevant lipid changes among different populations in this study and the speculation raised previously.

Two-pollutant model calculation found that the correlations between PM_10_ and PM_2.5_ exposures and adverse changes in blood lipid markers in the total hypertensive population and the hypertensive patients with arteriosclerosis remained basically stable after adjusting for the four types of gaseous pollutants respectively. Moreover, the blood lipid changes related to PM_10_ exposure were more significant than those of PM_2.5_ in the dual-pollutant model. Therefore, we believe that compared with gaseous pollutants, ambient particulate matter exposure is more strongly associated with increased TC, Lp(a) and decreased HDL-C, and the harmful effect of PM_10_ is greater than that of PM_2.5_. A longitudinal study in Shijiazhuang, China also suggests that ambient particulate matter may have a greater impact on lipid health than gaseous pollutants (49). In a hypertensive population with arteriosclerosis, the positive associations between ambient particulate matter exposure and HDL-C and LDL-C were partially reversed after adjustment for gaseous pollutants. Based on the results of the dual-pollutant model, we believe that ambient particulate matter exposure is not the main pollutant affecting the elevated HDL-C and LDL-C concentrations in this population. Combined with the previous conjecture, we suggest that the phenomenon of higher HDL-C associated with particulate matter exposure in people with hypertension without vascular stiffness, on the one hand, may be due to compensatory increases in HDL-C caused by the body's own protective mechanisms against lipid homeostasis. On the other hand, gaseous pollutants, which were positively correlated with each other and with particulate matter, exhibited stronger associations with HDL-C elevation. Based on the above results, we speculate that ambient particulate matter exposure can cause adverse changes in blood lipid levels in hypertensive patients and increase the risk of arteriosclerosis events.

This study has several strengths. First, we applied the detailed blood lipid detection data of inpatients collected by the hospital's big data center, and the real-time monitoring data of air pollution concentration collected by the environmental protection department to ensure the accuracy of the data source. We selected hypertensive patients as research subjects and grouped them according to whether they were accompanied by arteriosclerosis, so as to study the correlation between ambient particulate matter exposure and the risk of blood lipids in hypertensive patients with different disease states. Second, the data analysis applied a semiparametric generalized additive model. After adjusting for the effects of gender, age, weather, and time, the correlation between ambient particulate matter pollution and blood lipids in different lag time periods in a year was more accurately explored, and the relationship between each blood lipid index and air pollution exposure was calculated. Third, this study is the first to explore the relationship between air pollution and human blood lipid concentrations in southern Jiangxi, China, and its findings can provide reference for other regions with similar development levels and geographic latitudes.

This study also has some limitations. First, in addition to environmental factors, blood lipid levels are affected by a variety of factors, including genetics, behavior, and medication (50, 51). This study was not able to collect this information, so the results ignore the role of some valuable confounding factors and may be biased. Secondly, the air pollution concentration is the average of the data of the five monitoring points in Ganzhou City, and it is impossible to accurately understand the actual exposure concentration of air pollution of each research object. This exposed misclassification is likely to reduce the significance of the association (52). In addition, our study only analyzed the lipid effects associated with particulate matter exposure during a one-year period, and did not investigate the effect of a longer lag. Significant associations of LP (a) with ambient PM generally appeared at lag 359 days. Looking at the trends of LP (a) changes associated with exposure at different lag times, more significant associations with LP (a) increases may occur at longer exposure times.

## 5. Conclusions

Ambient particulate matter exposure was associated with higher TC, LP (a) and lower HDL-C in hypertensive patients, and PM_10_ exposure was more strongly associated with changes in lipid markers than PM_2.5_. There are association of long-term exposure to ambient particulate matter with the risk of arteriosclerosis in hypertensive patients, and such impacts have stronger harmful effects in patients with arteriosclerosis.

## Data availability statement

The raw data supporting the conclusions of this article will be made available by the authors, without undue reservation.

## Ethics statement

The studies involving human participants were reviewed and approved by the Ethics Committee of Gannan Medical University. The patients/participants provided their written informed consent to participate in this study. Written informed consent was obtained from the individual(s) for the publication of any potentially identifiable images or data included in this article.

## Author contributions

YG: conceptualization, writing–reviewing and editing, and funding acquisition. CL and LH: methodology, software, data curation, formal analysis, writing–original draft, review, and editing. XZho: software, formal analysis, investigation, and data curation. KH and MG: sensitivity analysis. XZha: conceptualization, data acquisition, supervision, and funding acquisition. All authors contributed to the article and approved the submitted version.
